# Supplementary figures and images for: Endorsement of reporting guidelines and clinical trial registration across urological medical journals: a cross-sectional study
Source: PeerJ. 2024 Dec 10;12:e18619. doi: 10.7717/peerj.18619 (PMC11639865; doi:10.7717/peerj.18619)

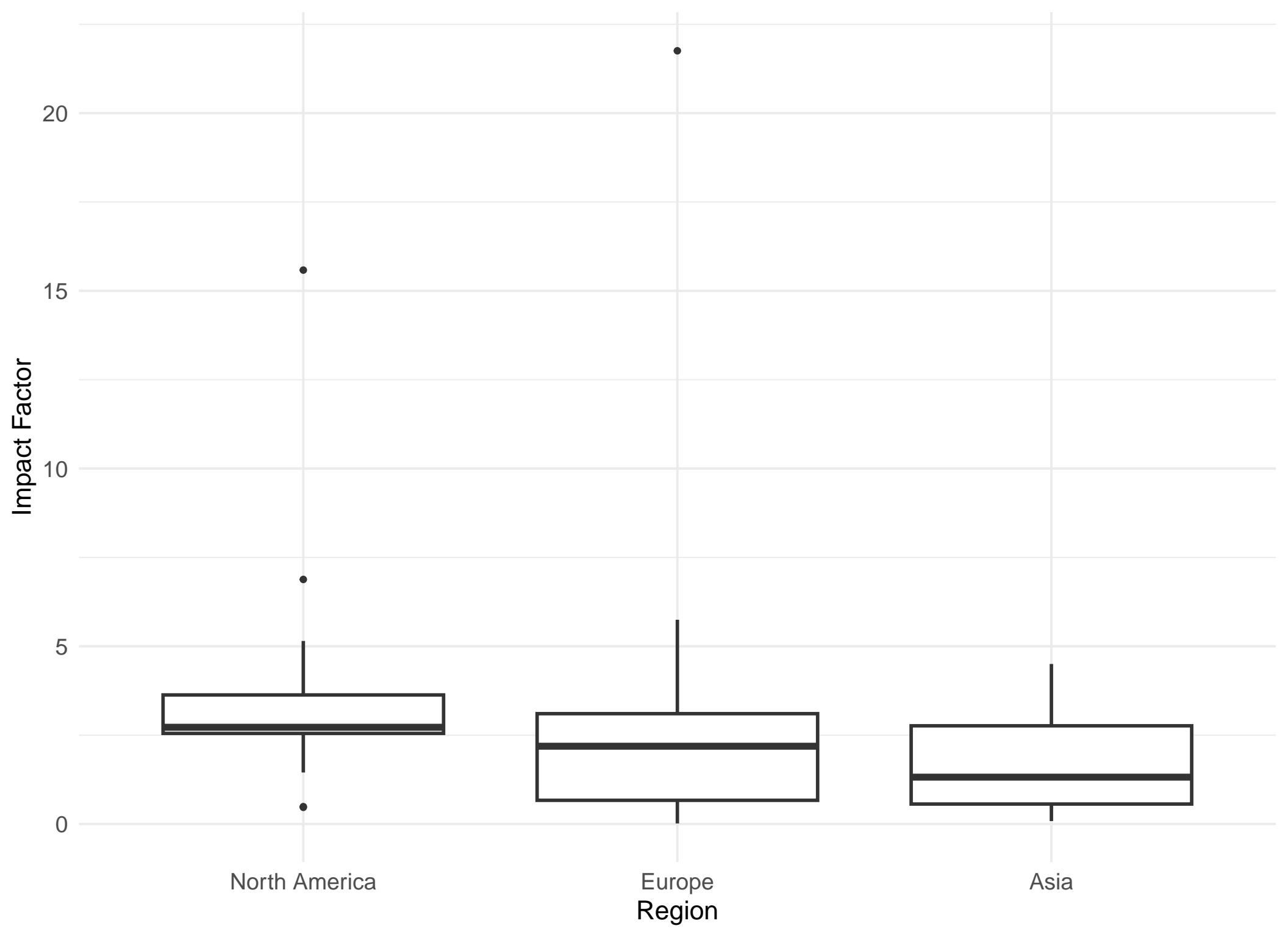

Supplement: Supplemental Information 1 — Box plot showing the distribution of urology journal impact factors by region: North America, Europe, and Asia. [file peerj-12-18619-s001.pdf]
